# Availability and Use of Essential Opioid Analgesics in Sub-Saharan Africa: A Scoping Review Protocol

**DOI:** 10.29337/ijsp.184

**Published:** 2023-02-01

**Authors:** Jane Yao, Ngo Valery Ngo, Odette D. Kibu, Constantine Asahngwa, Hilary M. Jasmin, Ronald M. Gobina, Denis A. Foretia

**Affiliations:** 1 College of Medicine, University of Tennessee Health Science Center, Memphis, TN, USA; 2 Division of Health Policy and Research, Nkafu Policy Institute, Yaoundé, Cameroon; 3 Department of Public Health, University of Buea, Cameroon; 4 Health Science Library, University of Tennessee Health Science Center, Memphis, TN, USA; 5 Buea Regional Hospital, Buea, Cameroon; 6 Center for Multicultural and Global Health, University of Tennessee Health Science Center, Memphis, TN, USA; 7 Global Surgery Institute, University of Tennessee Health Science Center, Memphis, TN, USA

**Keywords:** essential opioid analgesics, sub-Saharan Africa, Scoping Review Protocol, LMICs, opioid use, opioid availability

## Abstract

**Introduction:**

The treatment of acute, peri-operative, and chronic pain by healthcare practitioners and health systems requires appropriate access to and availability of essential opioid medications. While opioids are often oversupplied and overprescribed in high-income countries, there are significant inequities as many low- and middle-income countries (LMICs) experience severe shortages. In fact, while the richest 10% in the world reside in countries receiving almost 90% of all available opioids, 50% of the poorest in the world reside in countries receiving just 1% of all available opioids.

Understanding the social, economic, cultural, and regulatory barriers to access essential opioid analgesics in LMICs is critical in delineating and prioritizing appropriate interventions. We aim to conduct a scoping review on the availability and usage of essential opioid analgesics in LMICs, specifically in sub-Saharan Africa, to identify barriers, themes, and knowledge gaps.

**Materials and Methods:**

We will utilize the framework for conducting scoping reviews by Arksey and O’Malley. We will perform the search for articles in 3 electronic databases (i.e., SCOPUS, PubMed, Embase) and relevant gray literature. Only articles in English will be included. There will be no restriction on the publication period. All articles will directly involve either the availability and/or the use of essential opioid analgesics. Studies will be restricted to focus on sub-Saharan Africa. We will use a tailored extraction frame to extract relevant information from published articles that meet our inclusion criteria. We will analyze the data using both descriptive statistics and thematic analysis on the main study questions.

**Ethics and Dissemination:**

Since we will not be collecting primary data, formal ethical approval is not required.

Our study findings will be disseminated through abstracts, conference presentations, and peer-reviewed publications.

**Highlights:**

- 50 percent of the poorest in the world reside in countries receiving just 1 percent of all available opioids
- There is a paucity of data analyzing the inequitable distribution of essential opioid analgesics worldwide
- Our scoping review will identify barriers, themes, and knowledge gaps on the availability and use of essential opioids in SSA
- It will identify areas for further research and potential policy initiatives

## Introduction

Pain management associated with intra-operative and post-operative care requires appropriate supply and utilization of essential pain medications. The burden of post-operative pain for patients in low- and middle-income countries (LMICs), when compared to those in high-income countries (HICs), remains high, most notably in sub-Saharan Africa (SSA) [1]. Differences in utilization of essential opioid analgesics in the post-operative setting are contributing factors to this discrepancy. The distribution of opioid analgesics across the globe is highly inequitable [2]. While opioids are one of the most prescribed, used, and abused medicines in high-income countries (HICs) [3,4] many LMICs face severe shortages in accessing and prescribing opioids, including those deemed essential medicines for healthcare systems by the World Health Organization (WHO) [5]. There are significant disparities in patient outcomes between HICs and LMICs in areas such as acute and post-surgical pain management, chronic pain management, and palliative care for diseases, such as cancer [6]. Not only do LMICs have a higher burden of such diseases, but many also lack access to appropriate treatment options [7]. Opioids have been classified as essential pain medications by the World Health Organization since 1986, and the most up-to-date WHO Model List of Essential Medicines includes three essential opioid analgesics: morphine, codeine, and fentanyl [8]. Essential medicines included in the WHO’s core list are defined as “medicine needed for a basic healthcare system, listing the most efficacious, safe, and cost-effective medicines for priority conditions” [9].

Global morphine consumption remains inequitably distributed. Data from the WHO in 2003 indicated that six developed countries represented 79% of global morphine consumption [10]. Yet developing countries, making up 80% of the global population, represented just 6% of global morphine consumption [10,11]. This disparity has barely improved over the past decade. A 2021 study showed that consumption rates of opioid analgesics in LMICs have remained relatively stagnant over the study period from 2009 to 2019 [2]. Many challenges and barriers account for the shortage of opioids in LMICs. Key issues include inadequate supply and availability of prescription opioid analgesics in healthcare facilities, restrictive legislation and drug policy, lack of education and training on opioid prescribing for healthcare workers, and social/cultural perceptions regarding opioid use, misuse, and prescribing habits [12]. Studies have also demonstrated a direct correlation between physician density and opioid consumption rates. LMICs with lower physician densities had lower overall opioid consumption rates [2].

Existing literature reviews have focused very broadly on pain management and palliative care in LMICs [13,14]. Additionally, existing reviews have surveyed many LMICs from a global comparative perspective but have not focused on the landscape of opioid analgesics in the sub-Saharan Africa region [5,6]. SSA faces unique challenges regarding the supply chain for medications compared to even other LMICs. This includes an extremely weak manufacturing capacity for pharmaceuticals and various medications [15]. In addition, bulk purchasing of essential medications across the African continent, through mechanisms at the African Union, have been put in place to help address many of the supply-side challenges [16]. Despite the number of independent studies on specific pain management strategies in sub-Saharan Africa, there is little evidence that synthesizes the availability and/or utilization of essential opioid analgesics. The wide variation in opioid analgesics’ distribution and utilization globally—and specifically the disparities noted in LMICs—have strong implications for acute, peri-operative, and chronic pain management, especially palliative care. It is currently unclear what barriers exist in accessing essential opioid medicines in SSA and how shortages impact patient care and health outcomes. In identifying trends in utilization and challenges encountered, there is opportunity to identify global health and supply chain strategies that could be implemented to improve the accessibility and utilization of essential opioids in countries with major disparities.

Our scoping review proposes to understand opioid availability and use in Sub-Saharan Africa. We decided to focus this review on sub-Saharan Africa to better assess its unique challenges and evaluate potential mitigating strategies and policy options at national and continental levels. A scoping review was selected for this project to assess the breadth and depth of existing literature on the subject matter and to characterize the current landscape of specific opioid analgesics deemed as “essential medications” by the WHO. This scoping review will clarify knowledge gaps in the market availability and accessibility of essential opioid medicines and map out concepts pertaining to the economic, regulatory, and social barriers to accessing these medicines. Such thematic concepts, based on preliminary scoping of the literature, will potentially fall into the following themes: financing [17], knowledge and cultural beliefs [18], legislation and public policy [19], and education and training [20].

## AIMS of the Scoping Review

An initial search of the JBI journal for systematic reviews, the Cochrane Database of Systematic Reviews, CINAHL, and PROSPERO revealed no active, previous, or forthcoming scoping reviews on our proposed topic. Our overarching aim is to understand opioid availability and use in Sub-Saharan Africa and the current landscape of essential medications in LMICs. Specifically, our research will focus on

1. Mapping the evidence on the supply and utilization of essential opioid analgesics in sub-Saharan African countries
2. Identifying the social, economic, cultural, and regulatory barriers to accessing essential opioid analgesics in sub-Saharan Africa
3. Delineating appropriate interventions to prioritize to mitigate the situation

## Methodology

This scoping review will be guided by the five steps methodological framework proposed by Arksey and O’Malley [21]. This involves: (1) identifying the research question, (2) identifying relevant studies, (3) selecting eligible studies, (4) charting the data, and (5) collating and summarizing the results.

**Figure 1:**
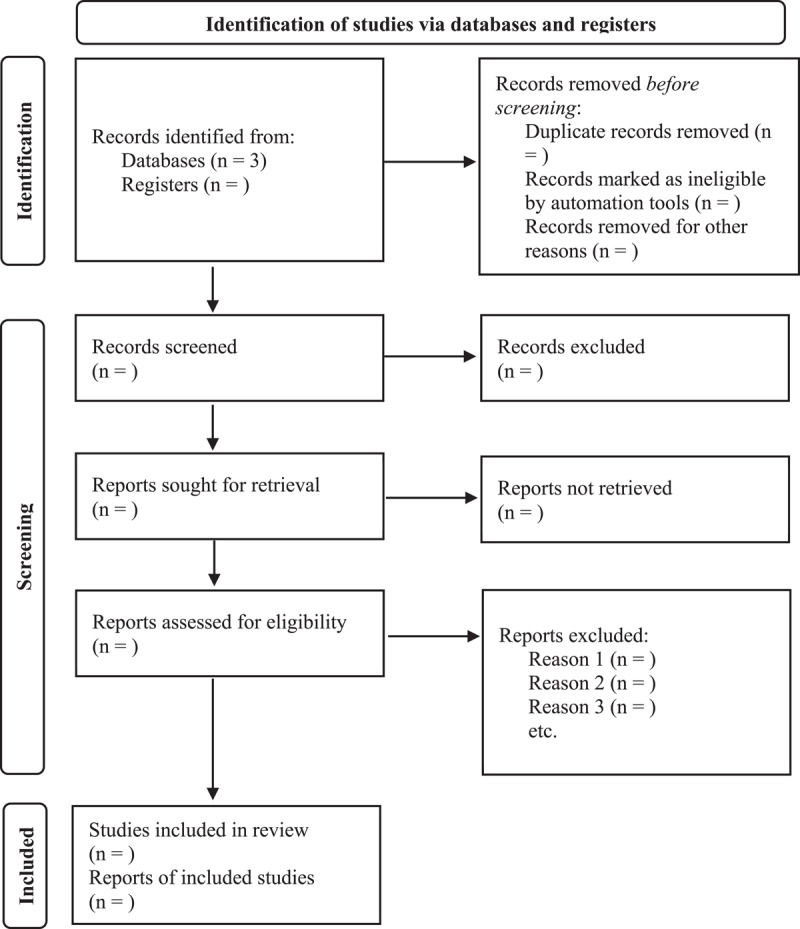
PRISMA Flow Diagram for Our Proposed Scoping Review [23].

This framework ensures that a straightforward methodological and transparent process is followed in examining the nature, range, and extent of research activities, as well as identifying knowledge gaps. The Preferred Reporting Item for Systematic Reviews and Meta-Analyses extension for Scoping Reviews (PRISMA-ScR) [22] will be utilized in our review for screening articles and reporting results. The PRISMA diagram is shown in Figure 1.

In lieu of a systematic review, a scoping review was chosen because the evidence relating to the availability and use of essential opioid analgesics in sub-Saharan Africa has not yet been comprehensively studied. Systematic reviews often address a single, specific research issue with established parameters, endpoints, and eligibility criteria for the included studies. In contrast, a scoping review might investigate many questions to identify potential patterns. Therefore, a scoping review is more appropriate for our proposed study, as it is currently unclear what the specific issues are regarding the availability and utilization of essential opioid analgesics in Sub-Saharan Africa.

### 1. Identifying the Research Question

To understand opioid availability and use in Sub-Saharan Africa, our research team will use an iterative process to look at the concept, target population, and outcomes as described by Arksey and O’Malley (Table 1).

**Table 1:** Arksey and O’Malley’s Concept, target group, and outcomes framework guiding our research questions.


Concept	Manufacturing and supply chain of essential opioid analgesics in Sub-Saharan Africa (SSA) National and international policies on the supply of essential opioids Cost of opioids in SSA Prescribing practices of healthcare personnel in SSA Knowledge gaps amongst healthcare personnel on the management of essential opioids

Target group	Sub-Saharan Africa Policymakers Healthcare practitioners Stakeholders involved in policy change

Outcome (the focus of papers)	Essential opioid (based on WHO classification 2021) Accessibility, availability, and barrier to access Overuse or abuse Economic and health policy


### 2. Identifying Relevant Studies

All team members, including the librarian, collaboratively developed, and agreed upon the search strategy, the inclusion and exclusion criteria, as well as the project timeline.

A preliminary search on the topic was conducted by the university librarian in PubMed. This was useful in helping us expand on search terms based on keywords, titles, and abstracts from the initial search. The detailed PubMed/MEDLINE database search strategy with search terms is shown in Appendix 1.

A systematic search will be conducted for published and unpublished (gray) literature using keyword combinations and Boolean operators “AND/OR”. MEDLINE via PubMed, SCOPUS and EMBASE will be exhaustively searched. Studies will be limited to sub-Saharan Africa, and there will be no restriction on publication dates. Search terms include methadone, morphine, fentanyl, and codeine, as well as general use of “opioid.” Additionally, every sub-Saharan African nation was used in searching. The search strategy will be piloted and checked for appropriateness of keywords and various databases. We will conduct a hand search of the reference list of all potentially relevant studies. Also, relevant gray literature will be identified through a targeted search of conference abstracts (EMBASE Conference Abstracts, Conference Proceeding, Africa Center for Disease Control, Africa Center for Evidence, and Africa Academy of Science), dissertations, and theses. The search result will be exported to Endnote for data management and to remove duplicates. Once duplicates are removed, the data will be exported to Rayyan (a free web tool designed to help researchers speed up the screening and selection of articles while working on systematic reviews, scoping reviews, and other knowledge synthesis projects) for screening.

#### Inclusion Criteria

The review will include articles published in English and that addresses the following: availability, accessibility, barriers to accessing essential opioids; assesses financial costs; evaluates economic and/or health policies; or looks at opioid prescription practices. Articles will be limited to Sub-Saharan Africa (as defined by World Bank). Primary, secondary, and observational research designs will be included.

#### Exclusion Criteria

Articles dealing with over the counter (OTC) analgesics, non-essential analgesics (as defined by WHO), or countries outside Sub-Saharan Africa will be excluded. Case reports, case series, editorials, animal models, genetic/molecular studies, clinical studies, and clinical trials will also be excluded.

### 3. Study Procedure and Selection of Relevant Studies

The titles and abstracts of all the articles exported to Rayyan will be double-blinded and screened by two independent reviewers (JY and VN) to select studies of relevance to our population, intervention, comparators, and outcomes (PICO) based on our inclusion and exclusion criteria.

Double screening of titles and abstracts will be checked independently by the reviewers (JY and VN) after unblinding, and unresolved conflicts will be sorted by a third reviewer (DF) who will decide on conflicting articles. The second stage will involve full-text screening, which will be done by JY, VN, OK, and CA, each working independently.

An attempt will be made to contact the authors of the articles without full text, while studies with multiple publications will have the latest of such publications retained. Unresolved conflicts between the reviewers will be resolved through discussion, and all conflicting articles will be arbitrated by a third reviewer (DF).

### 4. Data Extraction

Two independent reviewers will extract the data from full papers meeting all criteria. The Data Extraction Form in Table 2 will be used to collect relevant information from all included studies. Modifications will be made to the extraction form if necessary to capture unanticipated data points and emerging themes. All such modifications will be reported in the final scoping review. Where required, authors of publications with missing data will be contacted and the data requested.

**Table 2:** Data Extraction Form.


SPECIFIC FOCUS	DATA TO BE EXTRACTED	ADDITIONAL INFORMATION

General information	First author, year of publication, country, study design	

Study objectives	Aim of study/research questions	

Study participants	Description of population, characteristics of participants, sampling technique, and size	Exclude if the population is not from an LMIC in Sub-Saharan Africa

Methodology	Study design, setting, types of studies, method of data collection and analysis, etc.	

Outcomes	– Types of opioids available (name, classification, characteristics, safety, efficacy, etc.) – Opioids used for pain management (types, availability of choice, route of administration, health outcomes, etc.) – Accessibility (cost, perceptions, barriers, policy response, policy, practice gaps, etc.)	Exclude if the paper does not focus on an essential opioid (i.e., does not focus on morphine, fentanyl, or codeine)

	Does the paper address one or more of the following themes (pertaining to essential opioids): Financing Knowledge and Cultural Beliefs Legislation and Public Policy Education and Training Regional availability/supply (?)	If yes, refer to the respective following rows for details

Financing	Does the paper address the following: – Costs – Coverage and payment plans – Socioeconomic demographics	

Knowledge and Cultural Beliefs	Does the paper address the following: – Prescribing habits – Abuse/overdose rates	

Legislation and Public Policy	Does the paper address the following: – Policy on opioid supply and use – Regulation of opioid market supply and/or distribution – Prescribing protocol/guidelines for opioids	

Education and Training	Does the paper address the following: – Analgesic administration – Emergency overdose training – Acute vs. chronic vs. end-of-life care	

Local/regional availability and supply (?)	Does the paper address the following: – Presence of essential opioids in local/regional hospital networks, clinics, etc. – Specific availability/supply, e.g., vials, doses, supplies used for administration such as needles, etc.	


The extracted information will be recorded in a summary table to include the author’s information, study title, journal, year of publication, country or region of the study, the study objectives, and the study design. The concept or area addressed by the study in relation to our scoping review question will also be extracted. This will include: (1) Local/regional availability and supply, (2) consumption patterns, (3) costs and financing, (4) knowledge and cultural beliefs, (5) legislation and policy, (6) education and training, and (7) other themes emerging from the analysis.

### 5. Collating and Summarizing the Results

The aim of our scoping review is to collect existing evidence on the availability and use of essential opioid analgesics, as classified by the World Health Organization (WHO), in sub-Saharan Africa. We aim to summarize results from included studies into themes to identify current challenges and gaps in research.

The evidence will be summarized using descriptive statistics and thematic analyses. Descriptive statistics will include how many articles published, based on year, and involving either the availability and/or use of essential opioids in SSA. It will also involve country distribution of published studies. Our thematic analysis will focus on identified barriers to the availability and use of essential opioids with recommendations on how to mitigate them. The data will be manually extracted into key themes.

### 6. Data Presentation

Our extracted data will be displayed in diagrammatic or tabular format to serve the review’s goal. A narrative summary will accompany the diagrammatic or tabular findings in the results.

#### Ethics and Dissemination

Ethical approval is not required for this scoping review as data will be gathered by reviewing the current literature. Results of our study will be disseminated through abstracts, conference presentations, and peer-reviewed journal publications. Should we amend this protocol after publication, the date of the amendment and specific changes will be communicated in addition to the rationale for the change.

#### Patient and Public Involvement

No patient or public involvement in the design of this study.

## Discussion

Opioid analgesics are an important class of medications for the appropriate management of acute, peri-operative, and chronic pain. Worldwide distribution and consumption of opioids remains very inequitable with low- and middle-income countries, especially sub-Saharan Africa, significantly undersupplied [2,3,4]. Understanding the underlying drivers of this inequitable distribution is extremely important if appropriate mitigating strategies are to be implemented. Our proposed scoping review seeks to synthesize evidence and identify themes in the literature on the availability and utilization of essential opioid analgesics in sub-Sahara Africa. To our knowledge, this is the first scoping review of its kind in SSA.

We believe that this review will be a significant addition to the current literature on the barriers to accessing and utilizing essential opioids on the African continent. The results will be of interest to policy makers, clinicians, healthcare executives and pharmaceutical supply chain stakeholders in Africa and other parts of the world. The review will expand our understanding of the complexity and range of studies, identify themes, areas of possible stakeholder engagement and examples of successfully implemented interventions to increase access, availability, and use of essential opioids. The present study will also identify policy and research gaps at the national and continental level that will clarify areas for further research.

### Strengths and Limitations of Our Study

This review has major strengths as it includes all countries in sub-Saharan African and there are no date limits on the studies to be included in the review. This ensures we will be able to fully evaluate policy changes over a long timeframe and across the continent. However, it has some key limitations. By focusing on sub-Saharan Africa, the findings will not be generalizable to other low- and middle-income countries. Though the review may also be prone to publication bias, our use of a scoping review approach helps mitigate this by focusing on identifying themes. Another important limitation of our scoping review is that it will only look at data and articles published in English with a focus on sub-Saharan Africa. Therefore, studies in other languages, while equally important and even particularly insightful, will be excluded. In addition, relevant studies published after our review will be automatically excluded.

Despite these limitations, our study is particularly important and relevant as it will be the first to comprehensively review the published medical literature through major databases and with a focus on sub-Saharan Africa. Importantly, it provides guidance to investigators interested in conducting similar research in other settings.

## Appendix 1. PubMed/MEDLINE Search Strategy

((africa[MeSH Terms] OR sub saharan OR sub-saharan OR africa* OR Angola OR Benin OR Botswana OR Burkina Faso OR Burundi OR Cabo Verde OR Cameroon OR Central African Republic OR Chad OR Comoros OR Congo OR Cote d Ivoire OR Equatorial Guinea OR Eritrea OR Eswatini OR Swaziland OR Ethiopia OR Gabon OR Gambia OR Ghana OR Guinea OR Guinea-Bissau OR Kenya OR Lesotho OR Liberia OR Madagascar OR Malawi OR Mali OR Mauritania OR Mauritius OR Mozambique OR Namibia OR Niger OR Nigeria OR Rwanda OR Sao Tome OR Senegal OR Seychelles OR Sierra Leone OR Somalia OR South Africa OR South Sudan OR Sudan OR Tanzania OR Togo OR Uganda OR Zambia OR Zimbabwe) NOT (“guinea pig” OR “guinea-pig”))

AND

(codeine OR fentanyl OR methadone OR morphine OR “analgesics opioid”[Pharmacological Action] OR “analgesics, opioid”[MeSH Terms] OR opioid analgesi* OR opioid*).
